# Tris[2-(propyl­imino­meth­yl)phenolato-κ^2^ *N*,*O*]cobalt(III)

**DOI:** 10.1107/S1600536808014074

**Published:** 2008-05-17

**Authors:** Sheng Li, Shou-Bin Wang, Kun Tang, Yuan-Fang Ma

**Affiliations:** a The Institute of Immunology, Key Laboratory of Natural Drugs and Immunological Engineering of Henan Province, College of Medicine, Henan University, Kaifeng 475003, People’s Republic of China; b College of Chemistry and Chemical Engineering, Henan University, Kaifeng 475003, People’s Republic of China; c College of Medicine, Henan University, Kaifeng 475003, People’s Republic of China

## Abstract

The title compound, [Co(C_10_H_12_NO)_3_], was synthesized from cobalt(III) fluoride and 2-(propyl­imino­meth­yl)phenol in refluxing methanol. The Co^III^ ion is hexa­coordinated by three N and three O atoms from three bidentate Schiff base ligands in an octa­hedral geometry.

## Related literature

For related literature, see: Chung *et al*. (1971 ▶); Church & Halvorson (1959 ▶); Okabe & Oya (2000 ▶); Serre *et al.* (2005 ▶); Pocker & Fong (1980 ▶); Scapin *et al.* (1997 ▶).
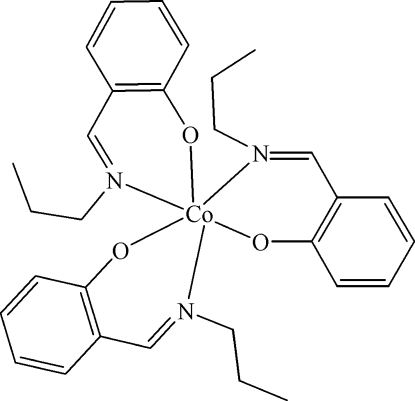


## Experimental

### 

#### Crystal data

[Co(C_10_H_12_NO)_3_]
*M* *_r_* = 545.55
Tetragonal, 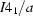

*a* = 19.588 (3) Å
*c* = 29.877 (6) Å
*V* = 11464 (3) Å^3^
*Z* = 16
Mo *K*α radiation
μ = 0.63 mm^−1^
*T* = 293 (2) K
0.43 × 0.28 × 0.22 mm

#### Data collection

Bruker APEXII CCD diffractometer
Absorption correction: multi-scan (*SADABS*; Bruker, 2001 ▶) *T*_min_ = 0.773, *T*_max_ = 0.873
41404 measured reflections
5133 independent reflections
3104 reflections with *I* > 2σ(*I*)
*R*_int_ = 0.075

#### Refinement

*R*[*F* ^2^ > 2σ(*F* ^2^)] = 0.047
*wR*(*F* ^2^) = 0.092
*S* = 1.00
5133 reflections
337 parameters
H-atom parameters constrained
Δρ_max_ = 0.29 e Å^−3^
Δρ_min_ = −0.22 e Å^−3^

### 

Data collection: *APEX2* (Bruker, 2004 ▶); cell refinement: *SAINT-Plus* (Bruker, 2001 ▶); data reduction: *SAINT-Plus*; program(s) used to solve structure: *SHELXS97* (Sheldrick, 2008 ▶); program(s) used to refine structure: *SHELXL97* (Sheldrick, 2008 ▶); molecular graphics: *SHELXTL* (Sheldrick, 2008 ▶); software used to prepare material for publication: *SHELXTL*.

## Supplementary Material

Crystal structure: contains datablocks I, global. DOI: 10.1107/S1600536808014074/cf2198sup1.cif

Structure factors: contains datablocks I. DOI: 10.1107/S1600536808014074/cf2198Isup2.hkl

Additional supplementary materials:  crystallographic information; 3D view; checkCIF report

## Figures and Tables

**Fig. 1.**
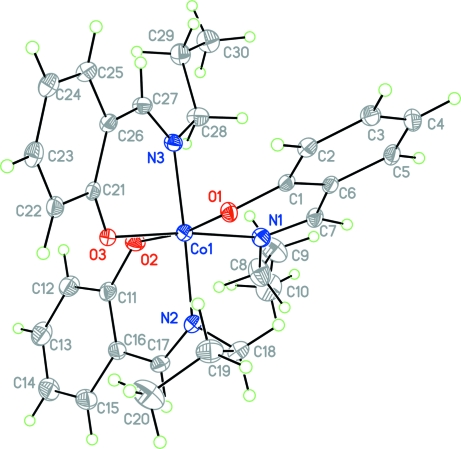
The molecular structure of (I), drawn with 30% probability displacement ellipsoids for the non-hydrogen atoms.

## supplementary crystallographic information

### Comment

In recent years, Schiff base ligands have been widely used as polydentate ligands, which can coordinate to transition or rare earth ions yielding complexes with interesting properties that are useful in materials science (Church & Halvorson, 1959) and in biological systems (Okabe & Oya, 2000; Serre *et al.*, 2005; Pocker & Fong, 1980; Scapin *et al.*, 1997). Here we report the synthesis and X-ray crystal structure analysis of the title compound, tris(*N*-n-propylsalicylaldiminato)cobalt(III).

The molecular structure of the title compound is shown in Fig.1. The Co^III^ ion is hexacoordinated by three N and three O atoms from three bidentate Schiff base ligands, in an octahedral geometry.  The Co—N and Co—O bond lengths are in the ranges 1.941 (2)–1.955 (2) and 1.8681 (19)–1.8999 (19) Å, respectively.

### Experimental

A mixture of cobalt(III) fluoride (0.5 mmol) and *N*-n-propylsalicylaldimine (0.5 mmol) in 40 ml methanol solution was refluxed for 5 h. The filtrate from the resulting soution was allowed to evaporate at room temperature for three days. Red crystals were obtained with a yield of 21%. Anal. Calc. for C_30_H_36_CoN_3_O_3_: C 65.99, H 6.60, N 7.70%; Found: C 65.91, H 6.53, N 7.64%.

### Refinement

All H atoms were placed in calculated positions with C—H = 0.93Å and refined as riding with *U*_iso_(H) = 1.2*U*_eq_(carrier).

### Crystal data

**Table:**

[Co(C_10_H_12_N_1_O_1_)_3_]	*Z* = 16
*M**_r_* = 545.55	*F*_000_ = 4608
Tetragonal, *I*4_1_/*a*	*D*_x_ = 1.264 Mg m^−^^3^
Hall symbol: -I 4ad	Mo *K*α radiation λ = 0.71073 Å
*a* = 19.588 (3) Å	Cell parameters from 5133 reflections
*b* = 19.588 (3) Å	θ = 1.2–25.3º
*c* = 29.877 (6) Å	µ = 0.63 mm^−^^1^
α = 90º	*T* = 293 (2) K
β = 90º	Block, red
γ = 90º	0.43 × 0.28 × 0.22 mm
*V* = 11464 (3) Å^3^	

### Data collection

**Table:**

Bruker APEXII CCD diffractometer	5133 independent reflections
Radiation source: fine-focus sealed tube	3104 reflections with *I* > 2σ(*I*)
Monochromator: graphite	*R*_int_ = 0.075
*T* = 293(2) K	θ_max_ = 25.3º
φ and ω scans	θ_min_ = 1.2º
Absorption correction: multi-scan(SADABS; Bruker, 2001)	*h* = −23→22
*T*_min_ = 0.773, *T*_max_ = 0.873	*k* = −23→23
41404 measured reflections	*l* = −35→35

### Refinement

**Table:**

Refinement on *F*^2^	Secondary atom site location: difference Fourier map
Least-squares matrix: full	Hydrogen site location: inferred from neighbouring sites
*R*[*F*^2^ > 2σ(*F*^2^)] = 0.047	H-atom parameters constrained
*wR*(*F*^2^) = 0.092	*w* = 1/[σ^2^(*F*_o_^2^) + (0.0331*P*)^2^] where *P* = (*F*_o_^2^ + 2*F*_c_^2^)/3
*S* = 1.00	(Δ/σ)_max_ < 0.001
5133 reflections	Δρ_max_ = 0.29 e Å^−^^3^
337 parameters	Δρ_min_ = −0.22 e Å^−^^3^
Primary atom site location: structure-invariant direct methods	Extinction correction: none

### Special details

**Table:**

Geometry. All e.s.d.'s (except the e.s.d. in the dihedral angle between two l.s. planes) are estimated using the full covariance matrix. The cell e.s.d.'s are taken into account individually in the estimation of e.s.d.'s in distances, angles and torsion angles; correlations between e.s.d.'s in cell parameters are only used when they are defined by crystal symmetry. An approximate (isotropic) treatment of cell e.s.d.'s is used for estimating e.s.d.'s involving l.s. planes.
Refinement. Refinement of *F*^2^ against ALL reflections. The weighted *R*-factor *wR* and goodness of fit *S* are based on *F*^2^, conventional *R*-factors *R* are based on *F*, with *F* set to zero for negative *F*^2^. The threshold expression of *F*^2^ > σ(*F*^2^) is used only for calculating *R*-factors(gt) *etc*. and is not relevant to the choice of reflections for refinement. *R*-factors based on *F*^2^ are statistically about twice as large as those based on *F*, and *R*- factors based on ALL data will be even larger.

### Fractional atomic coordinates and isotropic or equivalent isotropic displacement parameters (Å^2^)

**Table:**

	*x*	*y*	*z*	*U*_iso_*/*U*_eq_	
Co1	0.226327 (19)	0.48151 (2)	0.006993 (12)	0.05600 (15)	
C1	0.12898 (15)	0.41326 (14)	0.06122 (9)	0.0523 (7)	
C2	0.06948 (15)	0.37334 (14)	0.06292 (10)	0.0618 (8)	
H2	0.0562	0.3490	0.0376	0.074*	
C3	0.03038 (16)	0.36939 (16)	0.10098 (12)	0.0729 (9)	
H3	−0.0090	0.3429	0.1010	0.087*	
C4	0.04897 (19)	0.40445 (17)	0.13924 (11)	0.0760 (10)	
H4	0.0220	0.4019	0.1648	0.091*	
C5	0.10674 (18)	0.44248 (16)	0.13916 (9)	0.0674 (8)	
H5	0.1200	0.4648	0.1652	0.081*	
C6	0.14739 (15)	0.44903 (14)	0.10029 (9)	0.0547 (7)	
C7	0.20795 (17)	0.48916 (16)	0.10226 (10)	0.0664 (9)	
H7	0.2225	0.5026	0.1305	0.080*	
C8	0.3095 (2)	0.5453 (3)	0.07921 (14)	0.1220 (15)	
H8A	0.3371	0.5142	0.0968	0.146*	
H8B	0.3331	0.5516	0.0510	0.146*	
C9	0.3108 (3)	0.6025 (3)	0.0992 (2)	0.1486 (18)	
H9A	0.2888	0.5968	0.1281	0.235*	
H9B	0.2832	0.6343	0.0821	0.235*	
C10	0.3811 (2)	0.6360 (2)	0.10728 (15)	0.1317 (16)	
H10A	0.4146	0.6011	0.1124	0.198*	
H10B	0.3786	0.6653	0.1330	0.198*	
H10C	0.3937	0.6623	0.0815	0.198*	
C11	0.34024 (18)	0.54969 (19)	−0.03344 (10)	0.0704 (9)	
C12	0.36719 (19)	0.6075 (2)	−0.05589 (12)	0.0967 (12)	
H12	0.3405	0.6465	−0.0588	0.116*	
C13	0.4318 (2)	0.6068 (3)	−0.07339 (13)	0.1169 (15)	
H13	0.4482	0.6453	−0.0881	0.140*	
C14	0.4726 (2)	0.5506 (3)	−0.06963 (14)	0.1170 (16)	
H14	0.5162	0.5507	−0.0819	0.140*	
C15	0.44864 (19)	0.4935 (3)	−0.04758 (13)	0.1026 (13)	
H15	0.4767	0.4554	−0.0448	0.123*	
C16	0.38243 (17)	0.4920 (2)	−0.02915 (11)	0.0738 (9)	
C17	0.36155 (18)	0.43177 (19)	−0.00564 (10)	0.0754 (10)	
H17	0.3944	0.3979	−0.0025	0.090*	
C18	0.29465 (17)	0.35230 (18)	0.03437 (11)	0.0838 (10)	
H18A	0.2667	0.3585	0.0609	0.101*	
H18B	0.3391	0.3361	0.0440	0.101*	
C19	0.2619 (2)	0.29876 (19)	0.00453 (14)	0.1015 (12)	
H19A	0.2542	0.2580	0.0222	0.122*	
H19B	0.2176	0.3156	−0.0050	0.122*	
C20	0.3010 (2)	0.2796 (2)	−0.03554 (17)	0.1486 (18)	
H20A	0.3096	0.3195	−0.0533	0.223*	
H20B	0.2753	0.2472	−0.0528	0.223*	
H20C	0.3436	0.2595	−0.0267	0.223*	
C21	0.15472 (16)	0.43302 (15)	−0.06742 (9)	0.0538 (7)	
C22	0.14576 (18)	0.37797 (15)	−0.09769 (10)	0.0669 (9)	
H22	0.1829	0.3508	−0.1053	0.080*	
C23	0.0827 (2)	0.36426 (17)	−0.11598 (10)	0.0717 (9)	
H23	0.0781	0.3272	−0.1352	0.086*	
C24	0.02697 (18)	0.40319 (16)	−0.10686 (10)	0.0719 (9)	
H24	−0.0149	0.3937	−0.1202	0.086*	
C25	0.03369 (16)	0.45678 (16)	−0.07764 (10)	0.0674 (9)	
H25	−0.0041	0.4838	−0.0713	0.081*	
C26	0.09572 (16)	0.47160 (14)	−0.05731 (9)	0.0554 (7)	
C27	0.09995 (15)	0.52896 (15)	−0.02763 (10)	0.0632 (8)	
H27	0.0636	0.5595	−0.0284	0.076*	
C28	0.14549 (16)	0.60718 (16)	0.02585 (11)	0.0804 (10)	
H28A	0.1896	0.6293	0.0232	0.096*	
H28B	0.1396	0.5952	0.0571	0.096*	
C29	0.09207 (19)	0.65893 (16)	0.01440 (11)	0.0851 (10)	
H29A	0.0471	0.6390	0.0182	0.102*	
H29B	0.0970	0.6726	−0.0167	0.102*	
C30	0.0990 (2)	0.72063 (16)	0.04423 (12)	0.0996 (12)	
H30A	0.0863	0.7087	0.0743	0.149*	
H30B	0.0696	0.7563	0.0335	0.149*	
H30C	0.1455	0.7362	0.0439	0.149*	
N1	0.24463 (13)	0.50871 (13)	0.06881 (8)	0.0667 (7)	
N2	0.30289 (13)	0.41897 (13)	0.01157 (8)	0.0649 (7)	
N3	0.14848 (12)	0.54281 (12)	−0.00012 (8)	0.0595 (6)	
O1	0.16488 (9)	0.41360 (9)	0.02443 (6)	0.0589 (5)	
O2	0.27882 (10)	0.55441 (10)	−0.01683 (7)	0.0712 (6)	
O3	0.21526 (10)	0.44676 (10)	−0.05193 (6)	0.0622 (5)	

### Atomic displacement parameters (Å^2^)

**Table:**

	*U*^11^	*U*^22^	*U*^33^	*U*^12^	*U*^13^	*U*^23^
Co1	0.0519 (3)	0.0672 (3)	0.0489 (2)	0.0033 (2)	−0.00188 (19)	0.0004 (2)
C1	0.058 (2)	0.0494 (18)	0.0493 (18)	0.0086 (15)	−0.0039 (15)	0.0060 (14)
C2	0.065 (2)	0.060 (2)	0.060 (2)	0.0047 (17)	−0.0069 (17)	0.0039 (16)
C3	0.062 (2)	0.076 (2)	0.080 (2)	−0.0011 (18)	0.0054 (19)	0.020 (2)
C4	0.087 (3)	0.082 (3)	0.059 (2)	0.004 (2)	0.012 (2)	0.0108 (19)
C5	0.087 (3)	0.069 (2)	0.0460 (19)	0.009 (2)	−0.0033 (18)	0.0024 (15)
C6	0.064 (2)	0.0570 (19)	0.0434 (17)	0.0091 (16)	−0.0052 (15)	0.0019 (14)
C7	0.077 (2)	0.076 (2)	0.0454 (18)	0.0046 (19)	−0.0104 (17)	−0.0060 (17)
C8	0.127 (4)	0.149 (4)	0.089 (3)	−0.038 (3)	0.005 (3)	−0.034 (3)
C9	0.145 (4)	0.137 (4)	0.164 (5)	0.003 (3)	0.043 (4)	−0.037 (3)
C10	0.123 (4)	0.114 (3)	0.158 (4)	−0.065 (3)	−0.028 (3)	0.001 (3)
C11	0.057 (2)	0.093 (3)	0.061 (2)	−0.010 (2)	−0.0089 (18)	0.0071 (19)
C12	0.067 (3)	0.124 (3)	0.099 (3)	−0.012 (2)	−0.004 (2)	0.030 (2)
C13	0.075 (3)	0.169 (5)	0.107 (3)	−0.030 (3)	−0.002 (3)	0.050 (3)
C14	0.065 (3)	0.183 (5)	0.103 (3)	−0.014 (3)	0.017 (2)	0.025 (3)
C15	0.060 (3)	0.155 (4)	0.093 (3)	0.005 (3)	−0.002 (2)	−0.005 (3)
C16	0.052 (2)	0.108 (3)	0.062 (2)	0.002 (2)	−0.0023 (17)	−0.001 (2)
C17	0.064 (2)	0.095 (3)	0.067 (2)	0.021 (2)	−0.0136 (19)	−0.001 (2)
C18	0.079 (2)	0.090 (3)	0.082 (2)	0.021 (2)	−0.0080 (19)	0.019 (2)
C19	0.107 (3)	0.075 (3)	0.122 (3)	0.013 (2)	−0.001 (3)	−0.002 (2)
C20	0.145 (4)	0.137 (4)	0.164 (5)	0.003 (3)	0.043 (4)	−0.037 (3)
C21	0.066 (2)	0.0570 (19)	0.0383 (16)	0.0073 (17)	−0.0011 (15)	0.0092 (14)
C22	0.089 (3)	0.063 (2)	0.0491 (18)	0.0159 (19)	0.0059 (18)	0.0077 (16)
C23	0.103 (3)	0.060 (2)	0.0524 (19)	−0.006 (2)	−0.012 (2)	−0.0017 (15)
C24	0.086 (3)	0.067 (2)	0.063 (2)	−0.006 (2)	−0.0177 (18)	0.0015 (18)
C25	0.069 (2)	0.065 (2)	0.069 (2)	0.0059 (17)	−0.0114 (17)	0.0030 (18)
C26	0.063 (2)	0.0517 (18)	0.0515 (18)	0.0050 (16)	−0.0064 (15)	0.0026 (15)
C27	0.057 (2)	0.063 (2)	0.069 (2)	0.0084 (16)	−0.0028 (17)	−0.0052 (17)
C28	0.072 (2)	0.080 (2)	0.090 (2)	0.006 (2)	−0.0137 (19)	−0.023 (2)
C29	0.102 (3)	0.070 (2)	0.084 (2)	0.005 (2)	0.005 (2)	−0.0036 (19)
C30	0.125 (3)	0.063 (2)	0.110 (3)	0.003 (2)	0.007 (2)	−0.019 (2)
N1	0.0612 (17)	0.0784 (19)	0.0604 (17)	−0.0057 (14)	−0.0059 (14)	−0.0099 (14)
N2	0.0602 (17)	0.0818 (19)	0.0527 (15)	0.0094 (15)	−0.0065 (13)	0.0049 (14)
N3	0.0533 (15)	0.0673 (17)	0.0578 (16)	0.0018 (13)	−0.0015 (12)	−0.0110 (13)
O1	0.0628 (13)	0.0681 (13)	0.0459 (11)	−0.0031 (10)	0.0027 (10)	−0.0061 (10)
O2	0.0530 (13)	0.0765 (14)	0.0840 (15)	0.0001 (11)	0.0039 (11)	0.0079 (12)
O3	0.0566 (13)	0.0831 (14)	0.0468 (12)	0.0152 (11)	0.0020 (10)	0.0057 (10)

### Geometric parameters (Å, °)

**Table:**

Co1—O1	1.8681 (19)	C15—C16	1.409 (4)
Co1—O2	1.898 (2)	C15—H15	0.930
Co1—O3	1.8999 (19)	C16—C17	1.432 (4)
Co1—N2	1.941 (2)	C17—N2	1.283 (4)
Co1—N3	1.952 (2)	C17—H17	0.930
Co1—N1	1.955 (2)	C18—N2	1.482 (4)
C1—O1	1.305 (3)	C18—C19	1.519 (4)
C1—C2	1.405 (4)	C18—H18A	0.970
C1—C6	1.409 (4)	C18—H18B	0.970
C2—C3	1.373 (4)	C19—C20	1.470 (5)
C2—H2	0.930	C19—H19A	0.970
C3—C4	1.383 (4)	C19—H19B	0.970
C3—H3	0.930	C20—H20A	0.960
C4—C5	1.355 (4)	C20—H20B	0.960
C4—H4	0.930	C20—H20C	0.960
C5—C6	1.414 (4)	C21—O3	1.301 (3)
C5—H5	0.930	C21—C22	1.418 (4)
C6—C7	1.424 (4)	C21—C26	1.414 (4)
C7—N1	1.289 (3)	C22—C23	1.377 (4)
C7—H7	0.930	C22—H22	0.930
C8—C9	1.271 (5)	C23—C24	1.359 (4)
C8—N1	1.492 (4)	C23—H23	0.930
C8—H8A	0.970	C24—C25	1.372 (4)
C8—H8B	0.970	C24—H24	0.930
C9—C10	1.544 (6)	C25—C26	1.389 (4)
C9—H9A	0.970	C25—H25	0.930
C9—H9B	0.970	C26—C27	1.434 (4)
C10—H10A	0.960	C27—N3	1.286 (3)
C10—H10B	0.960	C27—H27	0.930
C10—H10C	0.960	C28—N3	1.482 (3)
C11—O2	1.305 (3)	C28—C29	1.497 (4)
C11—C12	1.418 (4)	C28—H28A	0.970
C11—C16	1.407 (4)	C28—H28B	0.970
C12—C13	1.369 (5)	C29—C30	1.508 (4)
C12—H12	0.930	C29—H29A	0.970
C13—C14	1.366 (5)	C29—H29B	0.970
C13—H13	0.930	C30—H30A	0.960
C14—C15	1.380 (5)	C30—H30B	0.960
C14—H14	0.930	C30—H30C	0.960
			
O1—Co1—O2	171.62 (8)	N2—C17—C16	127.8 (3)
O1—Co1—O3	85.97 (8)	N2—C17—H17	116.1
O2—Co1—O3	89.08 (9)	C16—C17—H17	116.1
O1—Co1—N2	91.64 (10)	N2—C18—C19	112.6 (3)
O2—Co1—N2	94.74 (10)	N2—C18—H18A	109.1
O3—Co1—N2	85.83 (9)	C19—C18—H18A	109.1
O1—Co1—N3	88.00 (9)	N2—C18—H18B	109.1
O2—Co1—N3	85.39 (9)	C19—C18—H18B	109.1
O3—Co1—N3	91.74 (9)	H18A—C18—H18B	107.8
N2—Co1—N3	177.56 (10)	C20—C19—C18	115.7 (3)
O1—Co1—N1	92.80 (10)	C20—C19—H19A	108.3
O2—Co1—N1	92.85 (10)	C18—C19—H19A	108.3
O3—Co1—N1	173.60 (9)	C20—C19—H19B	108.4
N2—Co1—N1	87.93 (10)	C18—C19—H19B	108.4
N3—Co1—N1	94.50 (10)	H19A—C19—H19B	107.4
O1—C1—C2	118.7 (3)	C19—C20—H20A	109.5
O1—C1—C6	123.9 (3)	C19—C20—H20B	109.5
C2—C1—C6	117.4 (3)	H20A—C20—H20B	109.5
C3—C2—C1	121.6 (3)	C19—C20—H20C	109.5
C3—C2—H2	119.2	H20A—C20—H20C	109.5
C1—C2—H2	119.2	H20B—C20—H20C	109.5
C4—C3—C2	120.7 (3)	O3—C21—C22	119.8 (3)
C4—C3—H3	119.7	O3—C21—C26	124.0 (3)
C2—C3—H3	119.7	C22—C21—C26	116.2 (3)
C5—C4—C3	119.5 (3)	C23—C22—C21	120.8 (3)
C5—C4—H4	120.3	C23—C22—H22	119.6
C3—C4—H4	120.3	C21—C22—H22	119.6
C4—C5—C6	121.5 (3)	C24—C23—C22	122.1 (3)
C4—C5—H5	119.3	C24—C23—H23	119.0
C6—C5—H5	119.3	C22—C23—H23	118.9
C5—C6—C1	119.4 (3)	C25—C24—C23	118.7 (3)
C5—C6—C7	119.0 (3)	C25—C24—H24	120.7
C1—C6—C7	121.5 (3)	C23—C24—H24	120.7
N1—C7—C6	126.6 (3)	C24—C25—C26	121.5 (3)
N1—C7—H7	116.7	C24—C25—H25	119.3
C6—C7—H7	116.7	C26—C25—H25	119.3
C9—C8—N1	122.6 (5)	C25—C26—C21	120.7 (3)
C9—C8—H8A	106.7	C25—C26—C27	119.0 (3)
N1—C8—H8A	106.7	C21—C26—C27	120.3 (3)
C9—C8—H8B	106.7	N3—C27—C26	127.1 (3)
N1—C8—H8B	106.7	N3—C27—H27	116.4
H8A—C8—H8B	106.6	C26—C27—H27	116.4
C8—C9—C10	117.7 (5)	N3—C28—C29	119.0 (3)
C8—C9—H9A	107.9	N3—C28—H28A	107.6
C10—C9—H9A	107.9	C29—C28—H28A	107.6
C8—C9—H9B	107.9	N3—C28—H28B	107.6
C10—C9—H9B	107.9	C29—C28—H28B	107.6
H9A—C9—H9B	107.2	H28A—C28—H28B	107.0
C9—C10—H10A	109.5	C28—C29—C30	110.2 (3)
C9—C10—H10B	109.5	C28—C29—H29A	109.6
H10A—C10—H10B	109.5	C30—C29—H29A	109.6
C9—C10—H10C	109.5	C28—C29—H29B	109.6
H10A—C10—H10C	109.5	C30—C29—H29B	109.6
H10B—C10—H10C	109.5	H29A—C29—H29B	108.1
O2—C11—C12	117.8 (3)	C29—C30—H30A	109.5
O2—C11—C16	124.4 (3)	C29—C30—H30B	109.5
C12—C11—C16	117.8 (3)	H30A—C30—H30B	109.5
C13—C12—C11	121.1 (4)	C29—C30—H30C	109.5
C13—C12—H12	119.4	H30A—C30—H30C	109.5
C11—C12—H12	119.4	H30B—C30—H30C	109.5
C14—C13—C12	121.1 (4)	C7—N1—C8	117.1 (3)
C14—C13—H13	119.4	C7—N1—Co1	123.3 (2)
C12—C13—H13	119.4	C8—N1—Co1	118.9 (2)
C15—C14—C13	119.6 (4)	C17—N2—C18	117.0 (3)
C15—C14—H14	120.2	C17—N2—Co1	122.7 (2)
C13—C14—H14	120.2	C18—N2—Co1	120.3 (2)
C14—C15—C16	121.1 (4)	C27—N3—C28	119.0 (2)
C14—C15—H15	119.4	C27—N3—Co1	121.2 (2)
C16—C15—H15	119.4	C28—N3—Co1	119.82 (19)
C15—C16—C11	119.2 (4)	C1—O1—Co1	125.85 (17)
C15—C16—C17	118.2 (4)	C11—O2—Co1	126.1 (2)
C11—C16—C17	122.6 (3)	C21—O3—Co1	120.51 (17)

## References

[bb1] Bruker (2001). *SAINT-Plus* and *SADABS* Bruker AXS Inc., Madison, Wisconsin, USA.

[bb2] Bruker (2004). *APEX2* Bruker AXS Inc., Madison, Wisconsin, USA.

[bb9] Chung, L., Rajan, K. S., Merdinger, E. & Crecz, N. (1971). *Biophys. J.* **11**, 469-475. 10.1016/S0006-3495(71)86229-X PMC1484009 5569493

[bb3] Church, B. S. & Halvorson, H. (1959). *Nature (London)*, **183**, 124–125. 10.1038/183124a0 13622720

[bb4] Okabe, N. & Oya, N. (2000). *Acta Cryst.* C**56**, 1416–1417. 10.1107/s0108270100012786 11118970

[bb5] Pocker, Y. & Fong, C. T. O. (1980). *Biochemistry*, **19**, 2045–2049. 10.1021/bi00551a006 6769470

[bb6] Scapin, G., Reddy, S. G., Zheng, R. & Blanchard, J. S. (1997). *Biochemistry*, **36**, 15081–15088. 10.1021/bi9719915 9398235

[bb7] Serre, C., Marrot, J. & Ferey, G. (2005). *Inorg. Chem.* **44**, 654–658. 10.1021/ic0487373 15679398

[bb8] Sheldrick, G. M. (2008). *Acta Cryst.* A**64**, 112–122. 10.1107/S0108767307043930 18156677

